# An Approach to Coordinate Efforts to Reduce the Public Health Burden of Stroke: The Delta States Stroke Consortium

**Published:** 2004-09-15

**Authors:** Virginia J Howard, Joe Acker, Camilo R Gomez, Sean R Orr, Ada H Griffies, Wanda Magers, Martha Phillips, James M Raczynski, John E Searcy, Richard M Zweifler

**Affiliations:** Assistant Professor of Epidemiology, School of Public Health, University of Alabama at Birmingham; Birmingham Regional Emergency Medical Services System, Birmingham, Ala; Alabama Neurological Institute, Birmingham, Ala; Alabama Neurological Institute, Birmingham, Ala; Max Michael III, MD, George Howard, DrPH, School of Public Health, University of Alabama at Birmingham, Birmingham, Ala; Mississippi State Department of Health, Jackson, Miss; University of Arkansas for Medical Sciences, Little Rock, Ark; University of Arkansas for Medical Sciences, Little Rock, Ark; Alabama Medicaid Agency, Montgomery, Ala; University of South Alabama, Mobile, Ala

## Abstract

Stroke is the third leading cause of death and a leading cause of disability in the United States, with a particularly high burden on the residents of the southeastern states, a region dubbed the "Stroke Belt." These five states — Alabama, Arkansas, Louisiana, Mississippi, and Tennessee — have formed the Delta States Stroke Consortium to direct efforts to reduce this burden. The consortium is proposing an approach to identify domains where interventions may be instituted and an array of activities that can be implemented in each of the domains. Specific domains include 1) risk factor prevention and control; 2) identification of stroke signs and symptoms and encouragement of appropriate responses; 3) transportation, Emergency Medical Services care, and acute care; 4) secondary prevention; and 5) recovery and rehabilitation management. The array of activities includes 1) education of lay public; 2) education of health professionals; 3) general advocacy and legislative actions; 4) modification of the general environment; and 5) modification of the health care environment. The Delta States Stroke Consortium members propose that together these domains and activities define a structure to guide interventions to reduce the public health burden of stroke in this region.

## Introduction

Stroke is the third leading cause of death and a leading cause of disability in the United States (1). Unfortunately, the burden of stroke does not fall proportionately on the nation's population. Residents of the southeastern states, a region dubbed the "Stroke Belt," carry a particularly high burden. The Stroke Belt has been defined on the basis of high rates of stroke mortality, but the causes of high stroke mortality are a matter of debate and uncertainty (2,3). Although the boundaries of the Stroke Belt are not distinct, eight southern states are considered to compose its core: North Carolina, South Carolina, Georgia, Tennessee, Alabama, Mississippi, Arkansas, and Louisiana.

**Figure 1:**
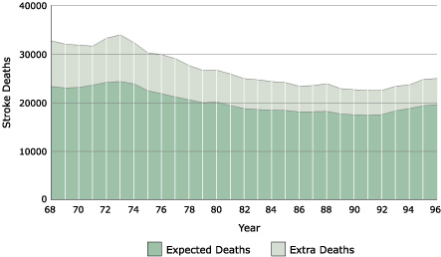
Number of annual deaths from stroke in North Carolina, South Carolina, Georgia, Tennessee, Alabama, Mississippi, Louisiana, and Arkansas, 1968–1996. The darker portion shows the number of deaths from stroke that would have occurred if the death rate from stroke were the same as for the remainder of the nation, while the lighter area represents the "extra" deaths above national rates.

The magnitude of the public health burden imposed by the Stroke Belt is overwhelming. Figure 1 shows the number of deaths from stroke in the eight-state region from 1968–1996. During this 29-year period, 780,385 total deaths resulted from stroke in this region. The expected number of deaths from stroke can be calculated by applying the national stroke death rate to the population of the region, resulting in an expected 585,836 total deaths from stroke during 1968–1996. The difference of 194,549 deaths represents the "extra" stroke deaths, or approximately 6708 extra deaths on average annually. Although stroke incidence data are not available, the extra number of incident stroke events in the region each year can be approximated by dividing the number of extra deaths each year (6708) by the case fatality rate (approximately 30%), resulting in 22,363 extra stroke events each year. The mean lifetime cost of ischemic stroke in the United States is estimated to be $140,048 (in 1999 dollars), which includes inpatient care, rehabilitation, and follow-up care (4). These data suggest that the annual public health burden imposed by the Stroke Belt is more than $3.1 billion dollars. (Note that this is not the burden of stroke in the region, but rather the extra costs associated with the increased stroke risk in the region.)

The Centers for Disease Control and Prevention (CDC) recently published *A Public Health Action Plan to Prevent Heart Disease and Stroke* (5), a comprehensive plan to reduce the burden of stroke and heart disease. One of the five major components of the proposed strategy is to encourage "engaging in regional and global partnerships [to] multiply resources and capitaliz[e] on shared experience" (5). The importance of developing partnerships in the southeastern United States to reduce the burden of stroke is evident, given the immense public health burden of stroke in the region. This need gave rise to the Tri-States Stroke Consortium, established in 1997 to coordinate the efforts of North Carolina, South Carolina, and Georgia (6). In 2002, the Delta States Stroke Consortium (DSSC) was formed to coordinate the efforts of the remaining five states in the Stroke Belt — Tennessee, Alabama, Mississippi, Arkansas, and Louisiana. This consortium includes representatives of state health departments, academic scientists, health care professionals, advocacy groups, pharmaceutical and other industry representatives, and stroke survivors. At the first meeting of the DSSC, held March 13–14, 2003, a plan for organizing efforts to reduce the burden of stroke in the region was developed and is summarized in this report.

## Identifying Opportunities to Reduce the Burden of Stroke

The DSSC developed a context for planning interventions to reduce the public health burden of stroke based on a two-dimensional model. The first dimension is based on the observation that stroke is not an event, but rather a process that begins with developing risk factors and continues through caring for stroke survivors. The second dimension represents the array of activities that can be implemented to reduce the burden of stroke. Each of these dimensions is summarized below.

### Domains in the process of stroke

The public health burden of stroke results from a process that begins in childhood (some would suggest prior to childhood), continues to adulthood, continues to the stroke event, and then to the subsequent care of the stroke survivor. The DSSC has divided this process into five domains. Within each domain, opportunities exist to reduce the burden of stroke.

**1. Risk factor prevention and control**

Prevention of stroke, as well as of most chronic diseases, has been shown to be the most cost-effective approach for reducing the public health burden of disease (7). The broad field of prevention is increasingly considered as being subdivided into two major domains: 1) primordial risk factor prevention and 2) risk factor control.

Primordial risk factor prevention, or preventing individuals from ever developing the risk factor, is clearly the best way to control the risk factor (8). Many risk factors for stroke, such as hypertension, diabetes, and obesity, have roots in childhood. Other risk factors, such as smoking, have roots in late adolescence. The first opportunity to reduce the burden of stroke is to intervene to reduce the development of risk factors.

There are, however, ample opportunities to reduce the burden of stroke after risk factors develop by improving the identification and control of those risk factors. For example, hypertension is the risk factor with the largest population-attributable risk: approximately 25% of strokes are attributable to the risk factor hypertension alone (9). While the number of hypertensive patients receiving appropriate diagnosis and management has improved dramatically, 31% of hypertensive patients are still unaware of their hypertension, and 69% of diagnosed hypertensive patients still do not control their condition adequately (10). Furthermore, benefits could be gained by better detection and control of other risk factors, including diabetes, atrial fibrillation, cigarette smoking, and other vascular risk factors (9,11,12).

**2. Identification of stroke signs and symptoms and encouragement of appropriate responses**

While some consider tissue plasminogen activator (t-PA) to be the only acute treatment for stroke, many other approaches, including hydration and blood pressure control, can improve the outcome of stroke and thereby reduce the subsequent burden of events. The effectiveness of these alternatives is supported by evidence showing that stroke patients have better outcomes when they receive stroke-unit care rather than general hospital care (13). However, the efficacy of these treatments is likely increased by the ability to intervene early during the stroke event. It is critical that the stroke is quickly identified and that it is perceived as a medical emergency that should be managed by professionals; hence, the burden of stroke can be reduced by improvements in the identification of strokes and in the decision making by the stroke victim and those witnessing the event. Specifically, it is critical that the public recognize stroke as a 911 emergency and that stroke victims be transported to the hospital as quickly as possible.

**3. Transportation, Emergency Medical Services (EMS) care, and acute care**

After the stroke is identified and 911 is contacted, the outcome of the stroke patient can be improved by prompt transport to an appropriate medical facility and delivery of appropriate care during the acute phase of the event. Effective transport is related to, but not solely determined by, the transport time from initial 911 call to emergency room delivery. Decisions must be made about the facility to which the patient should be taken and the kind of treatment that should be delivered during transport. In addition, the burden of stroke can be reduced by appropriate treatment after the patient arrives at the medical facility.

**4. Secondary prevention**

Stroke has a high rate of recurrence. The recurrence rate within 30 days for all cerebral infarcts in the Stroke Data Bank is 3.3%, and the one-year cumulative rate of death or recurrent infarction is 15.3% (14). Other studies have found the risk of recurrent stroke to be 8% in the first year and 12% after two years (15-17). Many first neurologic events have transient effects or minor long-term deficits; however, these patients are at elevated risk for subsequent major stroke. Many proven treatments reduce the subsequent risk of stroke, including risk factor management involving lifestyle changes, medical management, and surgical interventions (12,18).

**5. Recovery and rehabilitation management**

After a stroke has occurred, rehabilitation therapies can increase the stroke survivor's independence and quality of life, which have a direct impact on the quality of life of the survivor's family and caregivers and reduce the cost of post-stroke care.

### Array of activities to reduce the impact of stroke

The five domains discussed above provide opportunities to intervene to reduce the burden of stroke through an array of activities. The DSSC formed a working group for each domain to ensure that all opportunities and activities were considered. The Table shows a matrix that couples examples of activities with a specific domain. Clearly, certain activities may be more or less appropriate for each domain; however, use of this matrix ensures that all potential activities for each domain are considered.

A brief description of each general activity suggested by the DSSC is provided below.

**1. Education of lay public**

Perhaps the most promising of all activities to reduce the burden of stroke are efforts to educate the lay public. Educating the general public raises awareness of 1) lifestyle choices that lead to the development and control of risk factors, 2) stroke signs and symptoms, and 3) appropriate actions when signs and symptoms occur. Positive changes in lifestyle choices are associated with risk reduction. Education of the public also emphasizes the importance of obtaining and complying with rehabilitation efforts. The literature is rich with documentation of the lay public's lack of knowledge about the signs and symptoms of stroke (19-21), and there is an equally disturbing lack of knowledge in other domains such as risk factors (19,21), EMS care (22,23), and recovery and rehabilitation (24).

**2. Education of health care professionals**

Not only does the lay public lack knowledge about stroke prevention and care but health care professionals also have gaps in knowledge about opportunities to reduce the burden of stroke. Opportunities to improve the knowledge and training of health care providers include educating them about 1) lifestyle choices that prevent the development of risk factors; 2) better controls for existing risk factors; 3) appropriate guidance when initial signs and symptoms are reported; 4) actions that reduce the chances of subsequent strokes; and 5) potential gains offered by rehabilitation.

**3. General advocacy and legislative actions**

Another mechanism for reducing the burden of stroke is a highly focused effort for advocacy and legislative changes. Primordial risk factor prevention activities could include, for example, modification of public school lunches and urban design to encourage physical activity. An activity to promote primary control of risk factors could include public assistance for blood pressure medication. General advocacy activities could include the recruitment of lay opinion leaders to raise the awareness of stroke signs and symptoms. Legislative actions with an impact on the acute care of stroke patients should include encouraging the establishment of stroke centers (25). Finally, advocacy and legislative actions can reduce subsequent stroke and provide rehabilitation opportunities by ensuring access to services following the stroke event.

**4. Modification of the general environment**

Modifying the general environment is a potentially powerful tool in reducing the burden of stroke. Such activities include development of employee education programs, appropriate EMS signage, and home alterations to facilitate the return home of a stroke survivor.

**5. Modification of the health care environment**

Finally, there is the opportunity to modify the medical environment, including EMS transport, which should be designed to route stroke patients to hospitals equipped and ready to provide acute care as well as access to computed tomography (CT) imaging and rehabilitation services.

## Conclusions

The DSSC is organized into five working groups, with the emphasis of each group corresponding to one of the domains described in this report. The goal in defining these domains is to incorporate the entire spectrum of the stroke process, which places such a heavy burden on the United States, particularly in the southeastern states. Each working group developed an array of activities that have the potential to impact the public health burden of stroke.

Developing the list of potential activities in each of the domains, however, is only the first step. Each activity will be rated by a subcommittee both on its potential impact and the feasibility of its implementation. Subsequently, the DSSC aims to implement activities with a high potential impact and an acceptable feasibility in an ongoing effort to reduce the burden of stroke.

## Figures and Tables

**Table:** Matrix of Opportunities to Reduce the Burden of Stroke by Applying Activities Within Each of Five Domains, Delta States Stroke Consortium

		**Potential Activities to Reduce Stroke Burden**				

		**Education of lay public**	**Education of health care providers**	**Advocacy and legislative actions**	**Modification of general environment**	**Modification of health care environment**
**Domains for Interventions to Reduce the Burden of Stroke**	**Primordial and primary risk factor control**	Share resources and develop regional messages; partner with other organizations	Education of primary care providers	Advocate to provide reimbursement for provision of preventive care	Encourage environment for healthy lifestyle choices including walking paths and healthy snacks	Encourage systems for preventive care including standard assessments of lifestyle choices and risk factors
	**Identification of signs and symptoms, with appropriate actions**	Develop and disseminate a stroke first-aid course to the general public	Education of primary care physicians to immediately contact 911	Recruit panel of opinion leaders to assist in sending messages and raising awareness	Ensure complete 911 coverage	Train 911 operators on standard stroke identification and pre-transport care.
	**Stroke transport and acute care**	Develop and disseminate "Make the right call" and "Am I at risk?" programs	Develop and gain hospital adoption of uniform emergency room protocol for care of acute stroke	Reduce barriers to calling 911 and ensure that costs will be covered	Stress need for clear residential address identification for EMS	Develop a format and content for a bidirectional stroke transfer protocol to include type of transport, level of transport, and treatment prior to hospital arrival
	**Secondary stroke prevention**	Ensure that all hospitalized patients have education in risk factors for prevention of second strokes, signs and symptoms of stroke, and need for monitoring status	Ensure that the health care providers have adequate training to formulate an optimal secondary prevention plan on type of initial stroke or transient ischemic attacks	Work at the local and national level to increase awareness, funding, and quality control for secondary prevention by using AQAF/JCHCO standards for stroke centers	Encourage environment that facilitates control of risk factors	Improve efforts to provide quality home health care; improve education, communications, and staffing
	**Rehabilitation and recovery**	Design and develop a rehabilitation awareness course	Design and develop rehabilitation training programs for physicians and allied health professionals	Push for stroke recovery as a quality indicator (QI) for all federal programs	Foster development of patient and caregiver support groups	Utilize lifetime health programs, encourage contracts with local gymnasiums
